# Two possible etiologies of Guillain-Barré syndrome: mRNA-1273 (Moderna) vaccination and scrub typhus: A case report

**DOI:** 10.1097/MD.0000000000032140

**Published:** 2022-12-02

**Authors:** Byoung Wook Hwang, Jeong Bin Bong

**Affiliations:** a Department of Neurology, Chosun University School of Medicine, Gwangju, Republic of Korea.

**Keywords:** coronavirus disease 2019, Guillain-Barré syndrome, mRNA-1273 (Moderna) vaccination, scrub typhus

## Abstract

Guillain-Barré syndrome (GBS) is an acute inflammatory polyneuropathy related to infection with bacteria or virus and vaccination. Cases of GBS after coronavirus infection-19 (COVID-19) vaccination have been reported. However, cases of GBS after inoculation with mRNA-based COVID-19 vaccines, especially mRNA-1273, have rarely been reported compared to after inoculation with adenovirus vector-based COVID-19 vaccines. On 1 hand, GBS occurring after scrub typhus is often reported, but the exact pathological mechanism has not been elucidated. We report the case of a patient with GBS after inoculation with mRNA-1273 COVID-19 vaccine and scrub typhus.

**Patient concerns:**

A 47-year-old man received COVID-19 vaccination 4 weeks before admission. He had a fever, rash and general weakness 1 day after vaccination. After 3 weeks, the muscle strength of the extremities deteriorated to the extent that walking was impossible.

**Diagnosis, interventions, and outcomes:**

The patient developed quadriplegia with areflexia, axonal-type sensorimotor polyneuropathy was confirmed by nerve conduction study. The patient was diagnosed as GBS. Scrub typhus was also diagnosed as eschar was observed in the chest area and the serologic test of anti-R-tsutsugamushi antibody showed a strongly positive result. The patient received treatment with intravenous immunoglobulin at 0.4 g/kg daily for 5 days. Mechanical ventilation was applied during the intensive care unit. He was treated for scrub typhus simultaneously. Six months after the onset of the disease, the patient showed improvement to the point where he could work and exercise alone.

**Lessons:**

When GBS is suspected, early evaluation and treatment can lead to favorable outcomes. Considering that cases of GBS after COVID-19 vaccination have been reported, it is important to conduct early evaluation and management of patients with muscle weakness after COVID-19 vaccination to ensure early detection of GBS. And even if fever and rash are side effects that can occur frequently after vaccination, it is necessary to consider other diseases in addition to the side effects of the vaccine. This is to prevent delay in diagnosis and treatment of other diseases.

## 1. Introduction

Guillain-Barré syndrome (GBS) is a type of acute inflammatory polyneuropathy; it is characterized by limb weakness and accompanied by decreased deep tendon reflexes. In 60% to 70% of patients, various infections such as upper respiratory tract infection or enteritis precede the syndrome. Although it is difficult to identify the actual source of the syndrome in most cases, *Campylobacter jejuni* infection accounts for the highest number of cases, and it has been reported to occur even after infection with cytomegalovirus, Epstein-Barr virus, human immunodeficiency virus, and influenza virus.^[1]^ There are reports of GBS occurring after infection as well as after vaccination with a weakened toxin. Recently, with the worldwide spread of coronavirus disease 2019 (COVID-19), caused by severe acute respiratory syndrome coronavirus 2, various vaccines are being used to combat the disease, and cases of GBS as neurological side effects of such vaccinations have been reported.^[2]^

Here, we report a case wherein a history of COVID-19 vaccination and a prior scrub typhus were confirmed in a patient with GBS and present a literature review.

## 2. Case presentation

**Figure 1.**
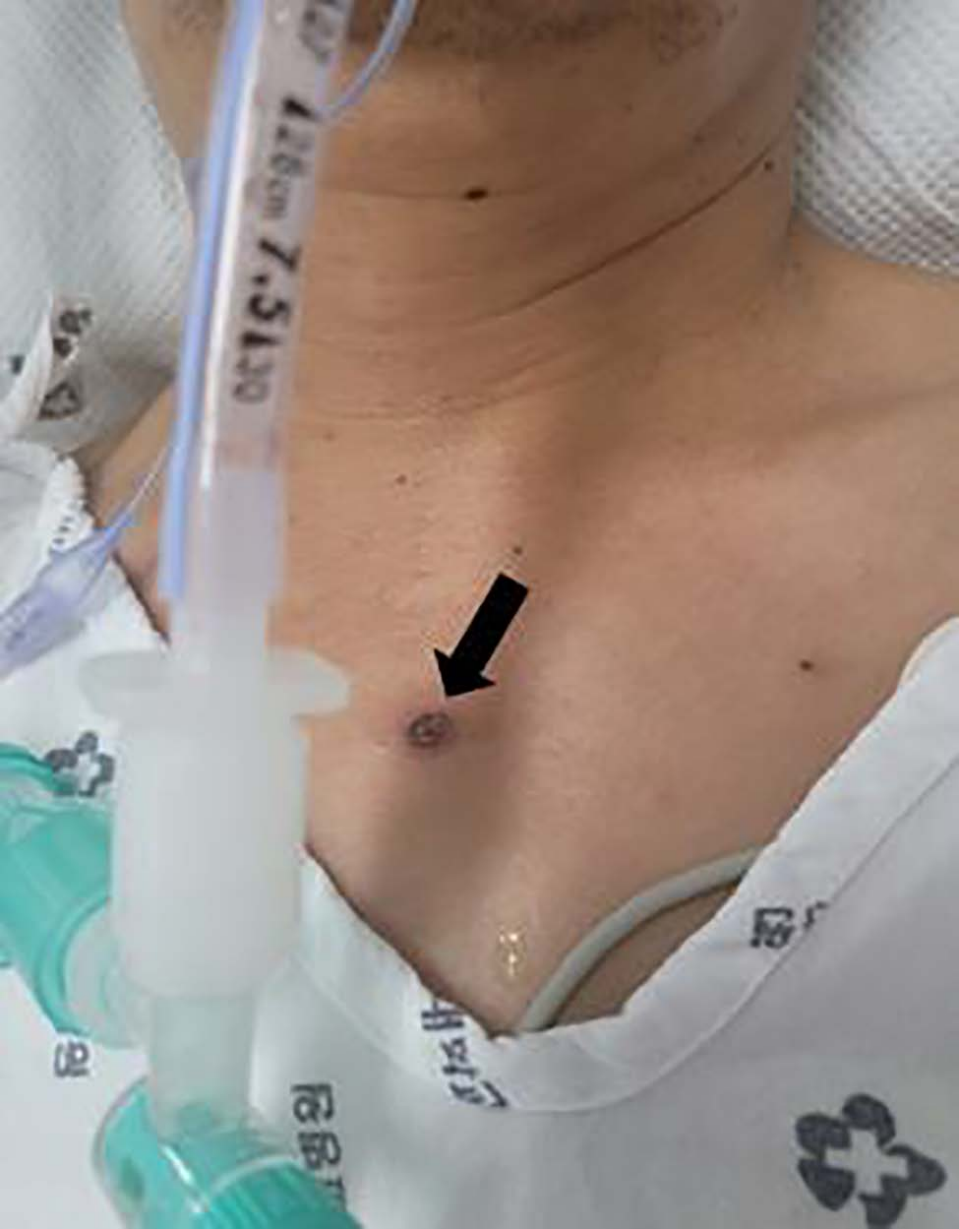
Photograph of the patient’s eschar on the anterior chest (arrow).

A 47-year-old man without any underlying disease visited our clinic with complaints of weakness in the lower extremities that occurred 4 days before admission. Four weeks ago, the first dose of the COVID-19 vaccine (mRNA-1273, Moderna) was administered, and a fever, general weakness, and generalized rash without pruritus appeared 1 day after vaccination. At primary care institution, these signs were judged to be side effects of the vaccination, and symptomatic treatment was implemented for approximately 3 weeks, but no improvement was reported. In the beginning, although there was a feeling of general weakness, the patient was able to walk and drive. However, the patient found difficulty in walking without the support of others at 4 days before our clinic visit and noted that it was impossible to walk on his own at 2 days before the visit. He also reported dyspnea on the day of admission to the emergency room. At the time of admission, his blood pressure and pulse were normal, but oxygen saturation could be maintained at 90% only when 3 L of oxygen was supplied through the nasal cavity. As a result of auscultation, no specific findings were noted for respiratory and heart sounds. On physical examination, a non-itchy rash was observed all over the body and an eschar on the chest was confirmed, and the eschar was first discovered by the medical staff after visiting the hospital (Fig. 1). On neurologic examination, his mental state was alert and cranial nerve function was normal. Bilateral lower extremity strength of Medical Research Council (MRC) grade III and bilateral upper extremity strength of MRC grade IV were observed. There was numbness on both sides below the wrist and below the ankle. Regarding the deep tendon reflexes, bilateral biceps reflex, knee reflex, and ankle reflex were absent, and pathological reflexes were not observed.

During a nerve conduction study performed on the day of admission, the compound muscle action potentials (CMAP) of the right ulnar nerve and median nerve were as low as 6.9 mV (normal range > 8 mV) and 2.3 mV (normal range > 6 mV), respectively, and the CMAP of the right peroneal nerve and tibial nerve were as low as 1.3 mV (normal range > 1.5 mV) and 0.9 mV (normal range > 6 mV), respectively. In the sensory nerve conduction study, the sensory nerve action potentials over finger-wrist segments of the right ulnar nerve and median nerve had decreased to 4.0 μV (normal range > 7.9 μV) and 7.5 μV (normal range > 8.8 μV), respectively. The F-wave of right peroneal nerve and tibial nerve was not observed, terminal latency was prolonged in all motor nerves, and almost all motor and sensory nerve conduction velocities were slightly decreased (Table 1).

**Table 1:** Results of nerve conduction study.

Nerve conduction parameters	Values	Normal value
Right ulnar nerve		
CMAP amplitude (mV)	**6.9**	>8
Distal latency (ms)	**3.6**	<2.51
Motor CV (m/s) (W-BE)	**40.4**	51.1–70.1
SNAP amplitude (μV)	**4.0**	>7.9
Sensory CV (m/s)	**35.1**	37.5–53.7
F-wave latency (ms)	29.2	<36
Right median nerve		
CMAP amplitude (mV)	**2.3**	>6
Distal latency (ms)	**5.3**	<3.9
Motor CV (m/s) (W-E)	**47.7**	50.5–68.1
SNAP amplitude (μV)	**7.5**	>8.8
Sensory CV (m/s)	**32.6**	39.3–55.3
F-wave latency (ms)	29.5	<33
Right peroneal nerve		
CMAP amplitude (mV)	**1.3**	>1.5
Distal latency (ms)	**6.4**	<5.3
Motor CV (m/s)	**40.1**	>40.5
F-wave latency (ms)	**absence**	<36
Right tibial nerve		
CMAP amplitude (mV)	**0.9**	>6
Distal latency (ms)	**7.4**	<5.4
Motor CV (m/s) (W-E)	**40.5**	>41.1
F-wave latency (ms)	**absence**	<36
Right superficial peroneal nerve		
SNAP amplitude (μV)	6.0	>2.0
Sensory CV (m/s)	**30.8**	>32.0
Right sural nerve		
SNAP amplitude (μV)	8.6	>6.0
Sensory CV (m/s)	**30.7**	>32.1

Abnormal values are bolded.

CMAP = compound muscle action potential, CV = conduction velocity, SNAP = sensory nerve action potential, W-BE = wrist-below elbow, W-E = wrist-elbow.

Cerebrospinal fluid (CSF) test revealed a leukocyte count of 16/mm^3^ (mono-dominant), protein level of 71.2 mg/dL, and glucose level of 51.2/110 (CSF/serum) mg/dL. At the time of admission, blood tests showed elevated white blood cells count of 17,840/mm^3^ (reference value, 4000–10,800/mm^3^) and C-reactive protein count of 8.61 mg/dL (reference value, 0–0.3 mg/dL), but infection focus was not observed on chest and abdominal computed tomography scans. Brain and whole spinal cord magnetic resonance imaging performed to exclude central nervous system lesions showed no specific findings.

Immunoglobulin was administered intravenously at a dose of 0.4 g/kg/d for 5 days with suspicion of GBS based on clinical symptoms and laboratory findings. Furthermore, in the additional medical history for the eschar observed at the time of admission to the emergency room, a history of outdoor activity of picking chestnuts in the mountains 1 month before admission was confirmed. In the serum indirect immunofluorescence antibody test, anti-R-tsutsugamushi antibody titer was confirmed to be 1:2560; scrub typhus was diagnosed, and oral doxycycline was administered at a dose of 200 mg/d for 5 days.

On the second day of hospitalization, the patient’s breathing difficulties worsened, so endotracheal intubation and mechanical ventilation was applied. On the third day of hospitalization, both upper and lower extremity muscle strength deteriorated to MRC grade I, and bilateral facial paralysis developed. Blood culture, urine culture, and sputum culture tests performed on the day of admission showed no specific findings. In addition, the results of serum virus antibody tests and CSF polymerase chain reaction tests including herpes simplex virus, varicella zoster virus, and Epstein-Barr virus, were all negative. Culture test results for bacterial, tuberculosis, and fungal infections of the CSF were also negative, and there were no specific findings for cytology. Both IgM and IgG-type anti-ganglioside antibodies (anti-GD1b, anti-GM1) test results were negative.

After using immunoglobulin for 5 days, symptomatic treatment was maintained, but muscle weakness in the upper and lower extremities continued on the 20th day of hospitalization, and mechanical ventilation was maintained. From the 26th day of admission, upper extremity muscle strength improved to MRC grade II, and on the 30th day of admission, the patient stopped using the mechanical ventilator, and he was discharged from the intensive care unit. At this time, in the follow-up nerve conduction study, the CMAP of the right peroneal nerve and tibial nerve were not measured, and the CMAP of the right median nerve and ulnar nerve were 1.7 and 2.2 mV, respectively, which were worse than the results obtained at the time of admission. As the muscle strength of the upper and lower extremities gradually improved, the patient was transferred to a rehabilitation hospital on the 52nd day of admission for active rehabilitation treatment. Four months after the onset, he was able to walk without assistance. And in follow-up nerve conduction study, the CMAP of the right median and ulnar nerves returned to normal values of 6.9 and 10.1 mV, respectively. And the sensory nerve action potentials of the right upper and lower extremities showed a trend of improvement, but the CMAP of the right peroneal nerve and tibial nerve were still not measured. Six months after the onset of the disease, he was able to work and exercise alone.

## 3. Discussion

Our patient developed fever, general weakness, and a generalized rash from the day after he received the COVID-19 vaccine (mRNA-1273; Moderna, Cambridge, Massachusetts). After 3 weeks, muscle weakness in the lower extremities rapidly worsened and he was admitted to the emergency room. At the time of admission to the emergency room, decreased muscle strength in the upper and lower extremities, loss of deep tendon reflexes, and decreased oxygen saturation were observed. And axonal-type sensory-motor polyneuropathy was observed in nerve conduction study, which was diagnosed as GBS. In addition, eschar was observed in the chest, and scrub typhus was also confirmed through blood test results.

GBS is an autoimmune disease of the peripheral nerves induced after bacterial or viral infection. *Campylobacter jejuni*, cytomegalovirus, Epstein-Barr virus, and *Mycoplasma pneumoniae* are the known causative agents according to case-control studies.^[3]^ In addition, not only prior infection but also vaccination, trauma such as surgery, and neoplastic disease are some of the known causes, but whether these antecedent events are related epidemiologically or temporally remains unclear.^[4]^ In the case of our patient, COVID-19 vaccination and history of scrub typhus can be considered the antecedents of GBS, but additional studies are required as these have not been definitively studied as antecedent events.

Scrub typhus is a disease caused by *Orientia tsutsugamushi*, which can proliferate in vascular endothelial cells and cause vasculitis throughout the body. Neurological complications can develop in about 20% of scrub typhus patients. Meningitis complications are well known, and other acute disseminated encephalomyelitis, cranial nerve damage, cerebral vascular disease including cerebral venous sinus thrombosis, transverse myelitis, nystagmus, and GBS have also been reported.^[5]^ There are several case reports of GBS occurring after scrub typhus.^[6–9]^ Unfortunately, however, no clear pathological mechanism has been elucidated other than the temporal relationship. However, since *Orientia tsutsugamushi* is known to induce both humoral and cellular immune responses,^[10]^ it is hypothesized that GBS is triggered by molecular mimicry similar to the known pathogenesis of GBS. In the case reports of 6 patients, 4 patients had eschar in the lower extremities (1 ankle, 3 knee), one in the axilla, and one in the scrotum.^[6–9]^ Facial palsy was also present in 3 patients. In nerve conduction study, 4 patients had an acute inflammatory demyelinating polyneuropathy pattern, one had an acute motor sensory axonal neuropathy pattern, and for 1 patient, a specific pattern was not described. Two patients required mechanical ventilation treatment due to respiratory failure, and all patients had good prognosis. Our patient also had facial palsy and required ventilator treatment due to respiratory failure but also had good prognosis, similar to findings in previous reports.^[8]^ The difference was that the pattern of polyneuropathy was acute motor sensory axonal neuropathy, in contrast to the acute inflammatory demyelinating polyneuropathy pattern that was mainly observed in the previous reports.

GBS after vaccination first received attention in 1976 when the incidence of GBS more than quadrupled after the swine influenza (A/New Jersey/1976/H1N1) vaccine.^[11]^ After the recent emergency approval of the COVID-19 vaccine, various studies have reported cases related to GBS confirmed after COVID-19 vaccination.^[12]^ However, the causal relationship between the COVID-19 vaccine and the occurrence of GBS has not yet been clearly established. In addition, many cases of GBS after vaccination with adenovirus vector-based vaccines such as ChAdOx1 nCoV-19 (Oxford/AstraZeneca) have been reported. However, there are some reports of GBS secondary to the mRNA-1273 vaccination, as seen in our patient’s case, emphasizing careful monitoring for GBS in all types of COVID-19-vaccinated patients. According to a study that reported on cases of GBS after COVID-19 vaccination, the period of onset of symptoms after the vaccination varied from 1 day to 3 weeks, but most patients developed symptoms within 2 weeks; mainly, polyneuropathy with demyelinating pattern was observed, and cell-albumin dissociation was observed in CSF tests. The patients also showed good prognosis after immunoglobulin treatment.^[2]^ In previous cases of GBS associated with the mRNA-1273 vaccine, such as in our patient, the symptoms of GBS developed immediately after the first inoculation in some patients, or there were no specific symptoms after the first inoculation, but the symptoms developed after the second one. One another case, mild symptoms occurred after the first inoculation, but GBS was not suspected at the time, and after the second inoculation, the symptoms worsened and GBS was finally diagnosed. Therefore, the number of vaccinations and the occurrence of GBS were not consistent. In addition, as in this case, gait disturbance due to weakness in the lower extremities occurred first in all 3 previous cases, and the general course of GBS was observed, where symptoms improved after immunoglobulin or plasmapheresis.^[13–15]^

Our patient had severe form of GBS accompanied by respiratory failure but recovered without serious neurological sequelae. The possible antecedents were COVID-19 vaccine and scrub typhus, indicating that both antecedent events should be considered in clinical settings. In addition, fever, general weakness, and rash symptoms that occurred 4 weeks before admission occurred the day after the COVID-19 vaccination; therefore, the primary medical institution judged it as a side effect of the vaccine and administered symptomatic treatment only. However, since scrub typhus, which was later confirmed, has fever, general weakness, and rash as the main symptoms, it is possible that the symptoms were mistaken for a side effect of the vaccine, leading to delay of scrub typhus diagnosis. Therefore, even if symptoms occur after vaccination, it is necessary to consider other diseases besides the side effects of the vaccine. And further studies and extensive investigations on the immunological and other correlations between the COVID-19 vaccine and GBS are needed.

## Author contributions

**Conceptualization:** Jeong Bin Bong.

**Data curation:** Byoung Wook Hwang.

**Supervision:** Jeong Bin Bong.

**Writing – original draft:** Byoung Wook Hwang.

**Writing – review & editing:** Jeong Bin Bong.
